# ﻿*Allium* cytogenetics: a critical review on the Indian taxa

**DOI:** 10.3897/CompCytogen.17.98903

**Published:** 2023-05-29

**Authors:** Biplab Kumar Bhowmick, Sayantika Sarkar, Dipasree Roychowdhury, Sayali D. Patil, Manoj M. Lekhak, Deepak Ohri, Satyawada Rama Rao, S. R. Yadav, R. C. Verma, Manoj K. Dhar, S. N. Raina, Sumita Jha

**Affiliations:** 1 Department of Botany, Scottish Church College, 1&3, Urquhart Square, Kolkata- 700006, West Bengal, India Department of Botany, Scottish Church College Kolkata India; 2 Department of Botany, University of Calcutta, 35 Ballygunge Circular Road, Kolkata- 700019, West Bengal, India University of Calcutta Kolkata India; 3 Angiosperm Taxonomy Laboratory, Department of Botany, Shivaji University, Kolhapur, Maharashtra- 416004, India Shivaji University Kolhapur India; 4 Amity Institute of Biotechnology, Research Cell, Amity University Uttar Pradesh, Lucknow Campus, Lucknow- 226028, Uttar Pradesh, India Amity University Uttar Pradesh Lucknow India; 5 Department of Biotechnology and Bioinformatics, North-Eastern Hill University, Shillong, Meghalaya- 793022, India North-Eastern Hill University Shillong India; 6 School of Studies in Botany, Vikram University, Ujjain, Madhya Pradesh 456010, India Vikram University Ujjain India; 7 Genome Research Laboratory, School of Biotechnology, University of Jammu, Jammu, Jammu and Kashmir- 180006, India University of Jammu Jammu India; 8 Amity Institute of Biotechnology, Amity University, Sector 125, Noida, Uttar Pradesh- 201313, India Amity University Noida India

**Keywords:** *Allium*, Chromosome, FISH, Genome size, Indian species, NORs, Telomere

## Abstract

The genus *Allium* Linnaeus, 1753 (tribe Allieae) contains about 800 species worldwide of which almost 38 species are reported in India, including the globally important crops (onion, garlic, leek, shallot) and many wild species. A satisfactory chromosomal catalogue of *Allium* species is missing which has been considered in the review for the species occurring in India. The most prominent base number is x=8, with few records of x=7, 10, 11. The genome size has sufficient clues for divergence, ranging from 7.8 pg/1C to 30.0 pg/1C in diploid and 15.16 pg/1C to 41.78 pg/1C in polyploid species. Although the karyotypes are seemingly dominated by metacentrics, substantial variation in nucleolus organizing regions (NORs) is noteworthy. The chromosomal rearrangement between *A.cepa* Linnaeus, 1753 and its allied species has paved way to appreciate genomic evolution within *Allium*. The presence of a unique telomere sequence and its conservation in *Allium* sets this genus apart from all other Amaryllids and supports monophyletic origin. Any cytogenetic investigation regarding NOR variability, telomere sequence and genome size in the Indian species becomes the most promising field to decipher chromosome evolution against the background of species diversity and evolution, especially in the Indian subcontinent.

## ﻿Introduction

The genus *Allium* Linnaeus, 1753 is considered a wonder crop of global importance, catering to the agriculture, condiment, pharmaceutical, nutraceutical and cosmetic sectors of economy owing to the presence of numerous species with tremendous significance. Among several herb species, an onion (*A.cepa* Linnaeus, 1753) that is valued throughout the continent attracts a lot of attention of the economic sectors mentioned above, followed by garlics, leeks and shallots having limited uses. Onion is the second of the five main world vegetables species (after tomato) whose worldwide production accounted for 9% of the total (42–45%) increase in production of vegetables between 2000–2019 (https://www.fao.org/3/cb4477en/online/cb4477en.html#chapter-2_1).

*Allium*, previously referred to Liliaceae, is now a member of Amaryllidaceae sensu Angiosperm Phylogeny Group or APG III (Haston et al. 2009). This large genus (about 800 species, Costa et al. 2020) was divided into 15 subgenera and 56 sections (Friesen et al. 2006). At present, *Allium* has its primary evolution centre across the Irano-Turanian phytochorion while secondary centres of diversity include Mediterranean basin and western North America (Friesen et al. 2006). The taxonomy and evolution of this diverse genus has been accepted as difficult.

Cytogenetics, being the only elementary discipline of genetics, focuses on genome structure, function and evolution. The evolutionary history of organisms is inscribed in the chromosomes, the physically visible form of genome. The very fundamental parameters such as chromosome count reports, when combined with molecular cytogenetic and phylogenetic data (Islam-Faridi et al. 2020; Senderowicz et al. 2021), or genome size estimates, can elucidate trends of evolution in context of ploidy changes. Molecular cytogenetic approaches, in line with the parameters mentioned already, can accelerate the understanding of the evolutionary questions (Borowska-Zuchowska et al. 2022; Nath et al. 2022). A general correlation between evolutionary trends and chromosomal features has been shown in many plant families (Van-Lume et al. 2017; Carta et al. 2020; Bhowmick and Jha 2022; Nath et al. 2022). Recently, a broad concurrence between karyology and geographical distribution has been shown in three Allioideae tribes, with respect to the diversification of Allieae to Northern Hemisphere from the Indian tectonic plate around 30 million years ago (Costa et al. 2020).

India is the world’s second-largest producer of onion after China, with a production rate of 16360 kg/ Ha (2020–2021) (https://eands.dacnet.nic.in/). After onion, *A.sativum* Linnaeus, 1753 (garlic) is the second largest species of *Allium* contributing significantly to agro-economical development of the country (https://eands.dacnet.nic.in/). Among the other species, *A.schoenoprasum* Linnaeus, 1753 and *A.roylei* Stearn, 1947 exhibited resistance qualities (Nanda et al. 2016) and promise adoption of advanced breeding. Keeping in mind the significance of *Allium* and the complications in taxonomy and evolution, a comprehensive summary of cytogenetic characters has been presented for Indian species of *Allium*.

## ﻿Data compilation

Distribution of taxa, chromosome counts, ploidy, karyotypes and molecular cytogenetic reports have been compiled from original publications, chromosome atlases and databases e.g. Database on Genome-Related Information of Indian Plants or d-GRIP (http://indianpcd.com/; Jha et al. 2019), Index to Plant Chromosome Numbers or IPCN (http://www.tropicos.org/project/ipcn, Goldblatt and Lowry 2011), Chromosome Counts Database or CCDB (http://ccdb.tau.ac.il/, Rice et al. 2015), The Plant DNA C-values database (https://cvalues.science.kew.org/, Pellicer and Leitch 2020) and Plant rDNA Database (www.plantrdnadatabase.com, Vitales et al. 2017). In case of synonyms, the present taxonomic designations are retained with appropriate references.

## ﻿Cytogenetic catalogue of *Allium* species in India

There are 35–40 species of *Allium* currently reported from India (ca. 38 species) (d-GRIP, Pandey et al. 2021, 2022). The species of *Allium* in India belong to nine subgenera namely, *Cepa* (5 species), *Allium* (5 species), *Amerallium* (4 species), *Reticulatobulbosa* (3 species), *Polyprason* (3 species), *Anguinum* (2 species), *Butomissa* (2 species), *Melanocrommyum* (1 species) and *Rhizirideum* (2 species) (Friesen et al. 2006). Majority of the *Allium* species prefer temperate mixed forests or rocky slopes ranging 1200–5480 meters of the western Himalayas (e.g. *A.atropurpureum* Waldst. et Kit., 1800, *A.atrosanguineum* Schrenk, 1842, *A.auriculatum* Kunth, 1843, *A.caesioides* Wendelbo, 1969, *A.carolinianum* Redouté, 1804, *A.consanguineum* Kunth, 1843, *A.fedschenkoanum* Regel, 1875, *A.griffithianum* Boiss., 1859, *A.loratum* Baker, 1874, *A.oreoprasum* Schrenk, 1842, *A.roylei*, *A.schoenoprasum* and *A.schrenkii* Regel, 1875). There are few species endemic to Kashmir and Uttarakhand (e.g. *A.gilgiticum* F.T. Wang et Tang, 1937 which is also endangered, *A.stracheyi* Baker, 1874 and *A.negianum* A. Pandey, K.M. Rai, Malav et S. Rajkumar, 2021) (Pandey et al. 2021). Rest of the species occupy the temperate habitats of north-eastern hill region (e.g. *A.fasciculatum* Rendle, 1906, *A.hookeri* Thwaites, 1864, *A.macranthum* Baker, 1874, *A.platyspathum* Schrenk, 1841, *A.prattii* C.H. Wright, 1903, *A.rhabdotum* Stearn, 1960, *A.sikkimense* Baker, 1874) while some wild or semi-wild species (*A.przewalskianum* Regel, 1875, *A.tuberosum* Rottler et Sprengel, 1825, *A.victorialis* Linnaeus, 1753, *A.wallichii* Kunth, 1843) occur in the western and eastern Himalayan regions.

## ﻿Chromosome counts

**Figure 1.**
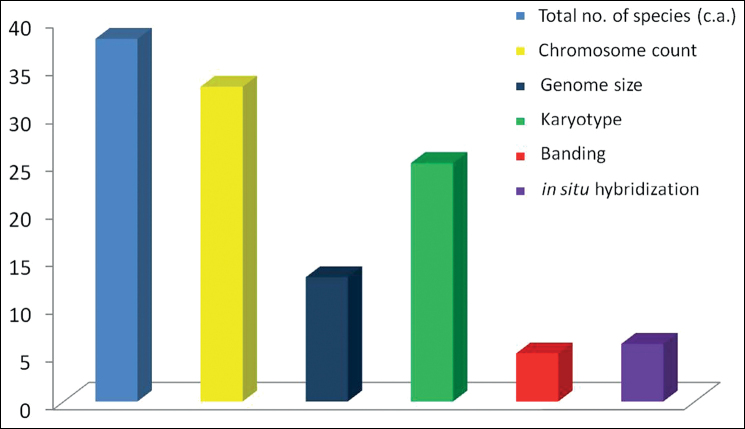
Bar graph showing statistics of cytological reports in the species of *Allium* in India.

The chromosome counts and karyotype details are known perhaps in 33 and 25 species, respectively (Table 1, Fig. 1). The prominent base number (x) is 8, irrespective of the subgenera, sections or the distribution pattern. Some western Himalayan species which are still not assigned to any of the subgenera (e.g. *A.atropurpureum*, *A.caesioides*, *A.consanguineum*, *A.ascalonicum* Linnaeus, 1756, *A.blandum* Wall., 1832, *A.hypsistum* Stearn, 1960) and endemic *A.stracheyi* have x=8. Divergent numbers such as x=7, 10 and 11 are found in the Indian species of the subgenus Amerallium (Table 1) which also justifiy their inclusion in a separate subgenus (Peruzzi et al. 2017). Chromosome number has not been studied in the newly discovered *A.negianum* of *Rhizirideum*, sect. Eduardia (Pandey et al. 2021), which together with its close relative *A.przewalskianum* of sect. Caespitosoprason (Pandey et al. 2021) not studied from the territory of India, needs to be investigated. Similarly, *A.loratum*, *A.auriculatum*, *A.rhabdotum* and an endemic *A.gilgiticum* still are not assigned to any of the subgenera, and any cytological information is also missing. The meiotic studies in some species have shown various configurations like multivalents or univalents and occasional irregularities as in *A.chinense* G. Don, 1827 (Gohil and Koul 1973, 1981), *A.hookeri* (Sharma et al. 2011), *A.roylei* (Sharma and Gohil 2003, 2011a; Kohli and Gohil 2011), *A.rubellum* M. Bieb., 1808 (Khoshoo and Sharma 1959; Koul et al. 1971) and *A.tuberosum* (Gohil and Koul 1983; Sharma and Gohil 2004, 2013a, b). In case of tetraploid *A.ampeloprasum* Linnaeus, 1753 (as *A.porrum* Linnaeus, 1753 in many studies), 16 bivalents were recorded regularly with complete absence of any multivalent (Koul and Gohil 1970b; Ved Brat and Dhingra 1973; Gohil and Koul 1977; Pandita and Mehra 1981a; Stack and Roelofs 1996). In this species, some peculiar features like appearance of bivalents in metaphase I instead of quadrivalents, localized chiasmata at pericentromeric regions have been reported (Levan 1940; Koul and Gohil 1970b; Stack and Roelofs 1996). Considering the incidence of vivipary and hybridization in *A.cepa* (Singh et al. 1967; Langer and Koul 1983; Puizina and Papea 1996), thorough meiotic analysis of the agriculturally important species (*A.cepa*, *A.sativum*, etc.) would be a significant aspect of future revision.

**Table 1.** Chromosome numbers, ploidy and nuclear genome sizes in Indian species of *Allium* of Amaryllidaceae (Tribe Allieae, Subfamily Allioideae, sensu APG IV 2016).

Subgenus/ section	Species (syn.)	Chromosome number			Ploidy	4C DNA value in diploid/ polyploid nuclei (pg)	Genome size in diploid/polyploid (pg)		References
		Basic (x)	Gametic (n)	Zygotic (2n)			1C	1Cx	
***Amerallium*/ *Bromatorrhiza***!	***A.fasciculatum* Rendle** (*A.gageanum*)	10^a^	–	20^b^, 40^c^	Diploid^d^, Tetraploid^e^	–	–	–	^a, b, d^(Xu et al. 1998; Li et al. 2017), ^b^(Huang et al. 1995), ^c, e^(Dutta et al. 2015)
*Amerallium*/ Bromatorrhiza*	***A.hookeri* Thwaites** (*A.tsoongii*)	–	–	22^a^, 33^b,^ 44^c^	–	63.24 (diploid, Feulgen cytophotometry)^d^	15.81 (diploid)^d^	15.81^d^	^a^(Sen 1974a; Tang et al. 2005; Sharma et al. 2011), ^a, b, c^(Huang et al. 1995), ^a, c^(Phuong et al. 2010), ^a, d^(Ohri et al. 1998; Ohri and Pistrick 2001)
***Amerallium*/ *Bromatorrhiza***!	***A.macranthum* Baker** (*A.oviflorum* Regel, *A.simethis* H.Lev.)	–	14^a^	14^b^, 28^c^	–	–	–	–	^a^(Levan 1934), ^b, c^(Huang et al. 1995; Tang et al. 2005)
*Amerallium*/ Bromatorrhiza*!	***A.wallichii* Kunth**. (*A.bulleyanum* Diels, *A.caeruleum* Wall.)	7^a^	-	14^b^, 28^c^, 32^d^	Diploid^e^, Tetraploid^f^	64.98 (diploid, Feulgen Cytophotometry)^g^, 121.79 (tetraploid, Feulgen Cytophotometry)^h^, 119.13 (tetraploid, Feulgen microdensitometry)^i^	16.24 (diploid)^g^, 30.45 (tetraploid)^h^	16.24^g^, 15.22^h^	^a, b, c, e, f^(Huang et al. 1995), ^c, f, i^(Labani and Elkington 1987), ^d^(Ved Brat 1965), ^a, b, c, e, f, g, h^(Ohri et al. 1998), ^a, b, c, e, g, i^(Ohri and Pistrick 2001)
*Anguinum*/ Anguinum*	***A.prattii* C.H.Wright** (*A.cannifolium* H. Lev., *A.ellipticum* Wall et Kunth)	8^a^	16^b^	16^c^, 32^d^	Diploid^e^, Tetraploid^f^	–	–	–	^a^(Lu et al. 2017), ^a, c, e^(Tang et al. 2005), ^b^(Kurosawa 1966), ^c, d, e, f^(Chunying et al. 2000)
***Anguinum*/ *Anguinum***!	***A.victorialis* L.** (*A.anguinum* Bubani, *A.reticulatum* St.-Lag.)	8^a^	8^b^	16^c^, 32^d^, 36^e^	Diploid^f^, Tetraploid^g^	81.00 (diploid)^h^, 86.42 (diploid, Feulgen microdensitometry)^i^, 162.02 to 167.10 (Tetraploid, Feulgen cytophotometry)^j^	20.25 (diploid)^h^, 21.60 (diploid)^i^, 40.5–41.78 (tetraploid)^j^	20.25^h^, 21.60^i^, 20.25–20.89	^a, b, f^(Pandita and Mehra 1981a), ^c, f^(Pandita and Mehra 1981b), ^a, c^(Mehra and Sachdeva 1976; Lu et al. 2017), ^c, f, i^(Labani and Elkington 1987), ^d, g, j^(Ohri et al. 1998), ^d, g, j^(Ohri and Pistrick 2001), ^e^(Sen 1973a), ^h^(Vakhtina et al. 1977)
***Melanocrommyum*/ *Brevicaule*** #	***Alliumchitralicum*** Wang & Tang (*A.badakhshanicum*, *A.pauli*)	–	–	16^a^, 32^b^	–	–	34.35 (tetraploid, flow cytometry)^c**^	17.17 (tetraploid, flow cytometry)^c**^	^a^(Pedersen and Wendelbo 1966), ^b, c**^(Gurushidze et al. 2012)
*Butomissa*/ Butomissa*	***A.tuberosum* Rottler ex Spreng.** (*A.chinense* Maxim., *A.clarkei* Hook.f.)	8^a^	8^b^, 16^c^, 32^d^	16^e^, 32^f^, 24^g^, 31, 33^h^, 48^i^, 61–64 ^j^, 62^k^, 64^l^	Tetraploid^m^, Hexaploid^n^, Octaploid^o^, Autotetraploid^p^, Autopolyploid^q^	66.80 (tetraploid)^r^, 121 (tetraploid)^s^, 109.36 (tetraploid, Feulgen cytophotometry)^t^, 121.47–123.25 (tetraploid, Feulgen Cytophotometry)^u^	30.36–30.62 (tetraploid)^u^	15.18–15.31^u^	^a, c, f, m, p^(Pandita and Mehra 1981a), ^a, f, m^(Talukder and Sen 2000; Kumar and Thonger 2018), ^b^(Li et al. 1985), ^c, f^(Sharma and Gohil 2004), ^c, p^(Sen 1974b), ^f, m, u^(Ohri et al. 1998; Ohri and Pistrick 2001), ^d^(Koul 1963), ^e^(Yang et al. 1998), ^f^(Sharma and Gohil 2013b; ^f, i, n, q^(Sharma and Gohil 2013a), ^g^(Huang et al. 1985), ^h^(Gohil and Koul 1973; Gohil and Kaul 1981), ^j^(Gohil and Kaul 1979; Ohri 1990), ^k^(Seo 1977), ^l^(Kojima et al. 1991), ^o^(Gohil and Kaul 1979), ^p^(Dutta and Bandyopadhyay 2014), ^r^(Nanushyan and Polyakov 1989), ^s^(Walters 1992), ^t^(Talukder and Sen 1999)
*Butomissa*/ Austromontana*!	***A.oreoprasum* Schrenk**	–	–	16 ^a^, 48 ^b^	–	–	–	–	^a^(Gohil and Koul 1973; Gohil and Kaul 1981), ^b^(Ved Brat 1965)
*Rhizirideum*/ Caespitosoprason*	***A.przewalskianum* Regel** (A.jacquemontiivar.parviflorum (Ledeb.) Aswal, *A.junceum* Jacquem. et Baker)	8^a^	–	16^b^, 32^c^, 64^d^	Diploid^e^, Tetraploid^f^ Octaploid^g^ Autopolyploid^h^	–	–	–	^a, b, e, f^(Tang et al. 2005), ^c^(Gohil and Kaul 1981), ^d, g^(Xue et al. 2000), ^h^(Ao 2008)
*Allium*/ Allium*	***A.ampeloprasum* L.** (*A.adscendens*, A.porrumvar.ampeloprasum)	8^a^	–	16^b^, 24^c^, 32^d^, 40^e^, 56^f^	Diploid^g^, polyploid^h^/ autotetraploid^i^	48.20 (tetraploid, feulgen cytophotometry)^j^, 100.54 (cytometry)^k^, 119.64/ 121.15 (tetraploid, feulgen cytophotometry)^l^, 119.80 (tetraploid, feulgen cytophotometry)^m^	16.7 (diploid, flow cytometry)^n**^, 25.35–27.45 (tetraploid, flow cytometry)^m,n**^	16.7 (diploid, flow cytometry)^n**^, 12.67–13.73 (tetraploid, flow cytometry)^m,n**^	^a, b, d, g, h, i, n**^(Ricroch et al. 2005), ^a, d, h, i^(Pandita and Mehra 1981a), ^c, e^(IPCN), ^d, h, i^(Maragheh et al. 2019), ^d, h, i, k^(Arumuganathan and Earle 1991), ^d, h, i, l^(Labani and Elkington 1987), ^d, h, i, m^(Ohri et al. 1998; Ohri and Pistrick 2001), ^f, h^(von Bothmer 1975), ^j^(Ranjekar et al. 1978)
*Allium*/ Allium*	***A.sativum* L.** (*A.arenarium* Sadler et Rchb, *A.controversum* Schrad. et Willd.)	8^a^	8^b^	16^c^, 12^d^	Diploid^e^	63.00 (diploid)^f^, 64.90 (diploid, Feulgen Cytophotometry)^g^, 65.40 (diploid)^h^, 66.40–69.00 (diploid)^i^, 68.20^l^, 71.40^m^, 73.59–91.80 (diploid, Feulgen Cytophotometry)^j^, 120 (diploid, Feulgen Cytophotometry)^k^	15.75 (diploid)^f^,16.23 (diploid)^g^,16.35 (diploid)^h^, 16.6–17.25 (diploid)^i^, 17.05^l^, 17.85^m^, 18.40–22.95 (diploid)^j^, 30.0 (diploid)^k^	15.75^f^, 16.23^g^, 16.35^h^, 16.6–17.25^i^, 17.05^l^, 17.85^m^, 18.40–22.95^j^, 30.0^k^	^a^(Gohil and Koul 1971), ^a, c, e^(Kumar and Thonger 2018), ^b^(Koul and Gohil 1970a), ^b, k^(Cortes et al. 1983), ^c, e^(Bacelar et al. 2021), ^c, e, g^(Ohri et al. 1998; Ohri and Pistrick 2001), ^d^(Sato and Kawamura 1981), ^h^(Ranjekar et al. 1978), ^f^(Murin 1976), ^i^(Chakravarty and Sen 1992), ^j^(Talukder and Sen 1999), l (Walters 1992), m(Olszewska and Osiecka 1982)
*Allium*/ Avulsea*	***A.griffithianum* Boiss.** (*A.bahri*, A.jacquemontiivar.grandiflorum)	8^a^	16^b^	16^c^, 32^d^	Diploid^e^, Tetraploid^f^, Autotetraploid^g^	41.15 (diploid, Feulgen cytophotometry)^h^	10.29 (diploid)^h^	10.29^h^	^a, b, d, f^(Pandita and Mehra 1981a), ^c, e, h^(Ohri et al. 1998; Ohri and Pistrick 2001), ^f, g^(Pandita and Mehra 1981b)
*Allium*/ Avulsea*	***A.rubellum* M. Bieb.** (*A.albanum* Grossh., *A.leptophyllum* Wall.)	–	16^a^	16^b^, 24^c^,	Diploid^d^, Triploid^e^, Tetraploid^f^, Numerical hybrid^g^, Autopolyploid^h^	–	–	–	^a, f^(Koul et al. 1971), ^b, d^(Abdali and Miri 2020), ^c^(Gohil and Koul 1973), ^e, h^(Khoshoo and Sharma 1959), ^g^(Ved Brat 1967)
***Allium*/ *Caerulea***!	***A.jacquemontii* Kunth**	8^a^	8^b^	16^c^	Diploid^d^	–	–	–	^a, b^(Pandita and Mehra 1981a), ^c^(Gohil and Kaul 1981), ^c, d^(Pandita and Mehra 1981b)
***Reticulatobulbosa*/ *Reticulatobulbosa***!	***A.humile* Kunth** (*A.govanianum*, *A.nivale*)	8^a^	8^b^	–	Diploid^c^	–	–	–	^a, b, c^(Pandita and Mehra 1981a), ^b^(Mehra and Sachdeva 1975), ^c^(Pandita and Mehra 1981b)
***Reticulatobulbosa*/ *Reticulatobulbosa***!	***A.schrenkii* Regel** (*A.bogdoicola* Regel)	–	–	32^a^	–	–	–	–	^a^(Friesen 1985)
*Reticulatobulbosa*/ Sikkimensia*	***A.sikkimense* Baker** (*A.kansuense* Regel, *A.tibeticum* Rendle)	–	–	16^a^, 32^b^	–	–	–	–	^a^(Mehra and Pandita 1979), ^b^(Gu et al. 1993)
*Polyprason*/ Falcatifolia*	***A.carolinianum* DC.** (*A.aitchisonii*, *A.obtusifolium*)	8^a^	16^b^	16^c^, 32^d^	Diploid^e^, Tetraploid^f^	52.90 (diploid, Feulgen cytophotometry)^g^	13.23 (diploid)^g^	13.23^g^	^a^(Tang et al. 2005), ^b^(Kumari and Saggoo 2016), ^c, e, g^(Ohri et al. 1998; Ohri and Pistrick 2001), ^d^(Gohil and Kaul 1981), ^f^(Oyuntsetseg et al. 2013), ^d, f^(Pandita and Mehra 1981b; Dutta et al. 2015)
*Polyprason*/ Oreiprason*	***A.roylei* Stearn** (*A.lilacinum* Royle et Regel, *A.rubens* Baker)	8^a^	8^b^	16^c^	Diploid^d^	63.00 (diploid)^e^, 70.03 (diploid, Feulgen microdensitometry)^f^	15.75 (diploid)^e^, 17.51 (diploid)^f^	15.75^e^, 17.51^f^	^a, b, c, d^(Kohli and Gohil 2011), ^b, c, d^(Sharma and Gohil 2011a; Kohli and Kaul 2013), ^c, d, e, f^(Labani and Elkington 1987), ^e^(Walters 1992)
*Polyprason*/ Falcatifolia*!	***A.platyspathum* Schrenk** (A.platyspathumsubsp.platyspathum)	–	–	16^a^	–	–	–	–	^a^(Friesen1986; Zakirova and Nafanailova 1988)
*Cepa*/ Cepa*	***A.cepa* L.** (A.cepavar.aggregatum, A.cepavar.anglicum)	8^a^	6^b^, 8^c^	14^d^, 16^e^, 24^f^	Diploid^g^, Triploid^h^	65.4 (diploid, flow cytometry)^i^, 66.40–69.00 (diploid, Feulgen cytophotometry)^j^, 67–71.61 (diploid, Feulgen cytophotometry)^k^, 67.5 (diploid, flow cytometry)^l^	16.35 (diploid)^i^, 16.60–17.25 (diploid)^j^, 16.75–17.90 (diploid)^k^, 16.87 (diploid)^l^, 16.2 (diploid)^m**^, 17.18–17.32 (diploid)^n**^	16.35^i^, 16.60–17.25^j^, 16.75–17.90^k^, 16.87^l^, 16.2^m**^, 17.18–17.32^n**^	^a, e, g^(Mancia et al. 2015), ^a, m**^(Ricroch et al. 2005), ^b^(Wang and Zheng 1987), ^c^(Ved Brat and Dhingra 1973; Gohil and Kaul 1980a; Talukder and Sen 2000; Sharma and Gohil 2011b), ^e^(Rees et al. 1979; Sato 1981; Joshi and Ranjekar 1982; Cortes et al. 1983; Schubert and Wobus 1985; Fuchs et al. 1995; Johnson and Zhatay 1996; Kim et al. 2002), ^e^(Van't Hof 1965) ^e, f, g, h^(Puizina and Papea 1996), ^e, g^(Narayan 1988; Ahirwar and Verma 2015), ^e, g, i^(Arumuganathan and Earle 1991), ^e, l^(Ulrich et al. 1988), ^e, j^(Chakravarty and Sen 1992), ^n**^(Baranyi and Grielhuber 1999), ^k^(Talukder and Sen 1999)
*Cepa*/ Annuloprason*	***A.atrosanguineum* Kar. et Kir.** (*A.monadelphum*)	8^a^	–	16^b^,32^c^	diploid^d^	–	–	–	^a, b, d^(Tang et al. 2005), ^b, d^(Ved Brat 1965), ^c^(Zhukova 1967)
*Cepa*/ Annuloprason*	***A.fedschenkoanum* Regel.** (A.atrosanguineumvar.fedschenkoanum)	8^a^	8^b^	16^c^	Diploid^d^	–	–	–	^a, b, d^(Pandita and Mehra 1981a), ^c, d^(Pandita and Mehra 1981b)
*Cepa*/ Sacculiferum*	***A.chinense* G. Don.** (*A.bakeri*, *A.bodinieri*)	8^a^	–	16^b^, 24^c^, 32^d^	Triploid^e^, Tetraploid^f^, Segmental allotetraploid^g^	130.86 (tetraploid, Feulgen cytophotometry)^h^	32.7 (tetraploid)^h^	16.35^h^	^a, d, f, i^(Ohri et al. 1998), ^a, d, f, g, h^(Ohri and Pistrick 2001), ^b^(Katayama 1928), ^c, d, e, f^(Wufeng et al. 1993), ^d^(Ohri et al. 1998; Ogura et al. 1999), ^d^(Dutta and Bandyopadhyay 2014), ^g^(Gohil and Koul 1981)
*Cepa*/ Schoenoprasum*	***A.schoenoprasum* L.** (*A.acutum* Spreng., *A.alpinum* (DC.) *Hegetschw*.)	8^a^	8^b^	14^c^, 16^d^, 24^e^, 32^f^, 48^g^	Diploid^h^	31.20 (diploid, 79)^i^, 33.20 (diploid)^j^, 33.80 (diploid)^k^, 34.90 (diploid)^l^ 37.73(diploid, Feulgen Cytophotometry)^m^, 60.66 (tetraploid)^n^	7.8 (diploid)^i^, 8.3 (diploid)^j^, 8.45 (diploid)^k^, 8.72 (diploid)^l^, 9.43 (diploid)^m^, 15.16 (tetraploid)^n^	7.8^i^, 8.3^j^, 8.45^k^, 8.72^l^, 9.43^m^, 7.58^n^	^a, b, h^(Pandita and Mehra 1981a), ^c^(Ohri 1990), ^d^(Dutta and Bandyopadhyay 2014), ^d, h, m^(Ohri et al. 1998; Ohri and Pistrick 2001), ^e^(Kurosawa 1979), ^f^(El-Gadi and Elkington 1977), ^g^(Pogosian 1997), ^h^(Pandita and Mehra 1981b), ^i^(Ranjekar et al. 1978), ^j^(Anderson et al. 1985), ^k^(Jones and Rees 1968), ^l^(Nanushyan and Polyakov 1989), ^n^(Labani and Elkington 1987)
–	***A.ascalonicum* L.** (*A.carneum*, *A.fissile*)	8^a^	8^b^	16^c^	Diploid^d^	66.32–68.67 (diploid, Feulgen cytophotometry)^e^	16.58–17.16 (diploid)^e^	8.29–8.28^e^	^a^(Darlington and Wylie 1955), ^b, c^(Cortes et al. 1983), ^d, e^(Talukder and Sen 1999)
–	***A.atropurpureum* Waldst. et Kit.** (A.nigrumvar.atropurpureum)	8^a^	8^b^	16^c^,32^d^	diploid^e^, tetraploid^f^	112.81 (tetraploid, Feulgen cytophotometry)^g^, 113.66 (diploid, Feulgen cytophotometry)^h^	28.2 (tetraploid)^g^, 28.45 (diploid)^h^	14.1^g^ 28.45^h^	^a, b, c, e^(Koul 1966; Pandita and Mehra 1981a), ^c, h^(Labani and Elkington 1987), ^d, f, g^(Ohri et al. 1998; Ohri and Pistrick 2001), ^c, h^(Gurushidze et al. 2012)
–	***A.blandum*** Wall.	–	16^a^	32^b^	Tetraploid^c^	–	–	–	^a, b, c^(Mehra and Sachdeva 1976; dGRIP)
–	***A.caesioides*** Wendelbo (*A.kachrooi*)	–	8^a^	16^b^	Diploid^c^	–	–	–	^a, b, c^(dGRIP) , ^a, c^(Pandita and Mehra 1981a), ^b^(Gohil and Kaul 1980a)
–	***A.consanguineum* Kunth**	8^a^	8^b^	16^c^	Diploid^d^	–	–	–	^a, b, d^(Pandita and Mehra 1981a), ^a, b^(Gohil and Koul 1971), ^c, d^(Gohil and Kaul 1980b)
–	***A.hypsistum* Stearn**	–	–	32^a^	–	–	–	–	^a^(dGRIP)
–	***A.stracheyi* Baker** (*A.longistaminum* Royle)	8^a^	8^b^	16^c^, 14^d^, 32^e^, 48^f^	Diploid^g^	–	–	–	^a, b, g^(Pandita and Mehra 1981a), ^c^(Pandita and Mehra 1981b), ^d^(Shopova 1966), ^d, e, f^(Sen 1974a)

*(Friesen et al. 2006), # (Fritsch et al. 2010), ! (Li et al. 2010), superscripts with the same letters correspond to references from which data are obtained, 1C and 1Cx genome sizes have been calculated from 4C DNA values published in references, ^**^ indicate 1C and 1Cx genome sizes that have been determined following 2C DNA values in corresponding references.

## ﻿Ploidy and genome size

The greatest variation in ploidy has been observed in *A.tuberosum* (subgenus Butomissa), *A.przewalskianum* (subgenus Rhizirideum), *A.chinense* G. Don, 1827 (subgenus Cepa) and *A.rubellum*, *A.ampeloprasum*, *A.griffithianum* (subgenus Allium) (Table 1). Polyploidy is reported in almost all subgenera and species. However, Peruzzi et al. (2017) reported absence of polyploidy in subgenus Anguinum and emphasized on correlation between chromosome size and ploidy to infer the trend of evolution. Any such correlation for Indian taxa is not possible at this stage due to lack of data for all the species.

Among the diploid species, the range of genome size (Table 1) is from 7.8 pg/1C in *A.schoenoprasum* (subgenus Cepa) to 30.0 pg/1C in *A.sativum* Linnaeus, 1753 (subgenus Allium). Among the polyploid taxa, the range of genome size (Table 1) is 15.16 pg/1C in *A.schoenoprasum*, 34.35 pg/1C (*A.chitralicum* F.T. Wang et Tang, 1937) to 40.5–41.78 pg/1C in *A.victorialis*. Thus, the lowest values of genome size for the entire array of *Allium* species in India is represented by diploid and polyploid species of *A.schoenoprasum* (subgenus Cepa).

The genome size evolution of *Allium* species has been envisaged in relation to growth pattern (dormancy), habitat preference and evolutionary history of the subgenera and sections (Ohri et al. 1998). The authors suggested an overall lack of correlation between genome size and chromosome numbers, although continuity in variation was particularly evident in few species. The present review has showed a 2.25-fold (diploid) or 2.43-fold (tetraploid) difference in genome size in the species occurring in India, although the base number (x) is predominantly 8.

## ﻿Karyotype features

The karyotype features are known in 8 subgenera and 14 sections of *Allium* species occurring in India (Fig. 1). The majority of species are characterized by metacentric chromosomes except for subgenus Amerallium with predominantly submetacentric chromosomes (Table 2). One pair of chromosomes with subterminal constriction has been the characteristic of some species such as *A.cepa* (Sato1981), *A.blandum*, *A.stracheyi* and *A.victoralis* (Mehra and Sachdeva 1976).

**Table 2.** Karyotype features and molecular chromosomal landmarks in species of *Allium* (Amaryllidaceae, Subfamily Allioideae, Tribe Allieae, sensu APG IV 2016) occurring in India.

Subgenera/ sections	Species	Karyotype		Heterochromatin banding (Giemsa/ Fluorochrome/others)	rDNA/ telomeric/ other signals		References
		Chromosome morphology	SAT or NORs/ 2n		No. of signals/2n	Features	
***Amerallium*/ *Bromatorrhiza***!	*A.fasciculatum* Rendle	Majorly submetacentric, few telocentric and metacentric^a^	4^b^	–	–	–	^a, b^(Xu et al. 1998; Dutta et al. 2015; Li et al. 2017)
*Amerallium*/ Bromatorrhiza*	*A.hookeri* Thwaites	Majorly submetacentric, few metacentric^a^	2^b^	–	–	–	^a, b^(Sharma et al. 2011)
*Amerallium*/ Bromatorrhiza*!	*A.wallichii* Kunth.	Majority submetacentric^a^	2^b^	–	–	–	^a, b^(Huang et al. 1995)
*Anguinum*/ Anguinum*	*A.prattii* C.H.Wright	Majority metacentric^a^	2^b^/4^c^	–	–	–	^a, b^(Tang et al. 2005), ^a, b, c^(Chunying et al. 2000)
***Anguinum*/ *Anguinum***!	*A.victorialis* L.	Majority metacentric^a^ or sub–metacentric^b^	2^c^	–	–	–	^a^(Pandita and Mehra 1981b), ^b, c^(Mehra and Sachdeva 1976)
*Butomissa*/ Butomissa*	*A.tuberosum* Rottler et Spreng.	Majority metacentric^a^ or submetacentric^b,c^	3^d^/ 4^e^ / 6^f^	–	5S:4–6^g^	5S: proximal and intercalary^h^	^a^(Kumar and Thonger 2018), ^a, e^(Talukder and Sen 2000), ^a, c, d, e^(Sharma and Gohil 2013b), ^a, b^(Sharma and Gohil 2013a), ^g, h^(Do and Seo 2000)
*Rhizirideum*/ Caespitosoprason*	*Alliumprzewalskianum* Regel	Majority metacentric chromosomes^a^	2^b^	–	–	–	^a, b^(Tang et al. 2005)
*Allium*/ Allium*	*A.ampeloprasum* L.	Majorly metacentric, few sub–metacentric^a^, few subacrocentric^b^	8^c^	Interstitial C– bands colocalized to silver stained regions in 8 active NORs^d^, 8 CMA3^+^/DAPI– bands colocalized to silver stained regions and 35S rDNA sites in NORs^e^	35S:8, 5S: 13 (polymorphic) ^f^	35S: interstitial (4) and pericentromeric (4) in short arms^g^, 5S: interstitial/ pericentromeric, non–coloclaized to 35S except in one chromosome of 8^th^ pair where it flanks 35S site^h^	^a, c, e, f, g, h^(Maragheh et al. 2019), ^b, c^(Koul and Gohil 1970b), ^b, c, d^(Stack and Roelofs 1996)
*Allium*/ Allium*	*A.sativum* L.	Majority metacentric^a^	2^b^/ 4^c^/ 6^d^/ 4–8^e^	C–Bands: nucleolar^f^, telomeric and interstitial^g^, centromeric (2 pairs)^h^; N–bands: nucelolar (4)^i^; Active NORs (AgNORs):2, occasionally4^j^; CMA^+^/DAPI–bands: 4–6^k^	5S: 4^l^, 6^m^; 45S and 5S rDNA localized^n^; telomeric signals in all chromosomes^o^; numerous satellite signals^p^	telomeric signals distal^q^, satellite signals sub–telomeric and interstitial^r^	^a^(Kumar and Thonger 2018), ^a, c^(Talukder and Sen 2000), ^a, k^(Bacelar et al. 2021), ^b^(Koul and Gohil 1970a), ^d, f, g^(Cortes et al. 1983), ^e, g, h, j^(Yuzbasioglu 2004), ^i^(Cortes and Escalza 1986; Wajahatullah and Vahidy 1990), ^l^(Do and Seo 2000), ^m^(Lee et al. 1999; Son et al. 2012), ^n^(Adams et al. 2001), ^o, p, q, r^(Peška et al. 2019)
*Allium*/ Avulsea*	*A.griffithianum* Boiss.	Majorly metacentric^a^		–	–	–	^a^(Pandita and Mehra 1981b),
*Allium*/ Avulsea*	*Alliumrubellum* M. Bieb.	Majority metacentric to sub–metacentric^a^	2^b^/ 6^c^/ 8^d^	–	–	–	^a, b^(Abdali and Miri 2020), ^a, c^(Koul et al. 1971), ^a, d^(Khoshoo and Sharma 1959)
***Allium*/ *Caerulea***!	*A.jacquemontii* Kunth	Majority metacentric^a^	–	–	–	–	^a^(Pandita and Mehra 1981b)
***Reticulatobulbosa*/ *Reticulatobulbosa***!	*A.humile* Kunth	Majority metacentric^a^	–	–	–	–	^a^(Pandita and Mehra 1981b)
*Polyprason*/ Oreiprason*	*A.roylei* Stearn	Majority metacentric^a^ or sub–metacentric^b^	2^c^	–	–	tyr-FISH mapping of bulb alliinase gene^d^	^a, b, c^(Sharma and Gohil 2008), ^c^(Kohli and Gohil 2011), ^d^(Khrustaleva et al. 2019)
*Polyprason*/ Falcatifolia*	*A.carolinianum* DC.	Majorly metacentric, few sub–meta– or sub–telocentric^a^	2^b^	–	–	–	^a, b^(Pandita and Mehra 1981b; Tang et al. 2005; Dutta et al. 2015;)
*Cepa*/ Cepa*	*A.cepa* L.	Majority metacentric, few submetacentric^a^	1^b^, 1–2^c^, 1–4^d^, 2^e^, 2–4^f^	C–Bands: telomeric^g^, intercalary^h^, distal^i^, centromeric and at satellites^j^; heterochromatic CMA/DAPI/AMD bands at NORs and telomeres^k^	18S–5.8S–25S rDNA loci: 2–4^l^, 45S rDNA loci: 3^m^, 4^n^, 4–5^o^, 5^p^; 5S rDNA loci: 2^q^, 4^r^	Variable rDNA sites^s^; distal 45S rDNA loci colocalized with telomeric tandem repeat^t^ and non–colocalized to 5S loci^u^; 5S loci proximal and distal^v^ or interstitial^w^; tyrFISH (with allinase, CHS–B and EST markers) reveal chromosome evolution^z^	^a^(Fiskesjo 1975; Sulistyaningsih et al. 2002; Ahirwar and Verma 2014), ^a, d, g^(Sato 1981), ^a,e^(Talukder and Sen 2000), ^a, f^(Battaglia 1957), ^b^(Bozzini 1964), ^c, g, h, j^(Puizina and Papea 1996), ^e,k^(Kim et al. 2002), ^e, o, q, s, u, w^(Mancia et al. 2015), ^g^(Ghosh and Roy 1977), ^i^(Tanaka and Taniguchi 1975), ^l^(Schubert and Wobus 1985), ^m^(Fu et al. 2019), ^n^(Do et al. 2001), ^p^(Ricroch et al. 1992), ^r^(Shibata and Hizume 2002), ^t^(Fajkus et al. 2016), ^v^(Shibata and Hizume 2002), ^z^(Khrustaleva et al. 2019)
*Cepa*/ Annuloprason*	*A.atrosanguineum* Kar. et Kir.	majorly metacentric^a^	2^b^	–	–	–	^a, b^(Tang et al. 2005)
*Cepa*/ Annuloprason*	*A.fedschenkoanum* Regel.	Majority metacentric chromosomes^a^	–	–	–	–	^a^(Pandita and Mehra 1981b)
*Cepa*/ Sacculiferum*	*A.chinense* G. Don.	Majority sub– metacentric^a^ or submetacentric^b^	2–4^c^	–	–	–	^a^(Ogura et al. 1999), ^a, c^(Sen 1973b), ^b, c^(Gohil and Koul 1981)
*Cepa*/ Schoenoprasum*	*A.schoenoprasum* L.	Majority metacentric^a^	1–6^b^	C–bands^c^	5S: 4^d^	5S: interstitial in chromosome 6^e^, tyr–FISH of alliinase reveal chromosome evolution^f^	^a^(Pandita and Mehra 1981b; Cai and Chinnappa 1987), ^a, b^(Dutta and Bandyopadhyay 2014), ^a, c^(Tardif and Morisset 1992), ^d, e^(Shibata and Hizume 2002), ^f^(Khrustaleva et al. 2019)
–	*A.ascalonicum* L.	metacentric to sub–metacentric^a^	2^b^	Distal C bands in all chromosomes^c^	–	–	^a, b^(Darlington and Haque 1955; Talukder and Sen 2000), ^b^(Darlington and Wylie 1955), ^b, c^(Cortes et al. 1983), ^c^(Seo and Kim 1975)
–	*A.atropurpureum* Waldst. et Kit.	Majorly nearly metacentric and few submetacentric^a^	–	–	–	–	^a^(Pandita and Mehra 1981b)
–	*A.blandum* Wall.	metacentric^a^	–	–	–	–	^a^(Mehra and Sachdeva 1976; Pandita and Mehra 1981b)
–	*A.consanguineum* Kunth	Majority metacentric or sub–metacentric chromosomes^a^	2 (interstitial)^b^	–	–	–	^a^(Mehra and Sachdeva 1976), ^a, b^(Pandita and Mehra 1981b)
–	*A.stracheyi* Baker	Majority metacentric^a^ or sub–metacentric^b^	–	–	–	–	^a^(Pandita and Mehra 1981b), ^b^(Mehra and Sachdeva 1976)

*(Friesen et al. 2006), # (Fritsch et al. 2010), ! (Li et al. 2010), superscripts with the same letters correspond to references from which data are obtained.

The predominance of metacentric chromosomes and symmetric nature of karyotypes is in accordance with earlier studies (Peruzzi et al. 2017). However, few species show a tendency for asymmetry (*A.atrosanguineum*, *A.carolinianum*, *A.griffithianum*, *A.fasciculatum*) and some fall into 2A (*A.chinense*, *A.przewalskianum*) or 2B category (*A.schoenoprasum*, *A.tuberosum*) in Stebbins’ index.

Presence of B-chromosomes has been reported in 97 species of *Allium* (Vujošević et al. 2013) belonging mostly to *Allium*, *Cepa* and *Rhizirideum* subgenera (Peruzzi et al. 2017). Among the species found in India, Sharma and Iyengar (1961) first reported the occurrence of B-chromosomes (2–10 in number) in diploid population of *A.stracheyi* and not in the polyploid populations. The B-chromosomes were found to occur in pollen mother cells as well as in pollen grains of *A.stracheyi* (Sen 1974c). However, Mehra and Sachdeva (1976) reported 2n=16 in *A.stracheyi* collected from the Valley of Flowers with no B-chromosome. One or two B-chromosome(s) were reported in *A.ascalonicum* (Bartolo et al. 1984), *A.ampeloprasum*, (subgenus Allium) (Khazanehdari and Jones 1996), *A.prattii* (subgenus Anguinum) (Chunying et al. 2000), *A.przewalskianum* (subgenus Rhizirideum) (Ao 2008; Xie-Kui et al. 2008) while many B-chromosomes (1–10) were recorded in *A.schoenoprasum* (Halkka 1985; Cai and Chinnappa 1987; Tardif and Morisset 1992) and in *A.stracheyi* (subgenus Cepa) (Sharma and Aiyangar 1961; Shopova 1966; Pandita and Mehra 1981b).

Nucleolus organizer regions or NORs are significant markers for chromosome identification. Among the species considered presently, NORs/ satellite-bearing chromosomes often show infra-specific or cultivar-specific differences particularly in *A.cepa*, *A.sativum* and *A.tuberosum* (Table 2).

In case of subgenus Allium, eight active NORs have been shown in *A.ampeloprasum* by C- banding, CMA3^+^/DAPI- banding, AgNOR staining and FISH (Table 2). In *A.sativum* secondary constrictions were observed in two to even six chromosomes by C and N banding (Ghosh and Roy 1977; Roy 1978; Cortes et al. 1983), in addition to showing population specific differences (Roy 1978). NORs were also confirmed in four chromosomes by N banding (Cortes and Escalza 1986; Wajahatullah and Vahidy 1990). Recently, two pairs of chromosomes with secondary constrictions were reported in some Brazilian accessions of *A.sativum* of which one pair was suggested to contain intercalary NOR (Bacelar et al. 2021). CMA banding method was used to show the infraspecific heterochromatin variability of nucleolar (proximal) and non-nucleolar (distal and proximal) CMA bands in the Brazilian garlic accessions for their identification. This study remains to be done in case of Indian cultivars.

*Alliumcepa* varieties with different ploidy levels (e.g. A.cepavar.viviparum, then supposed to be a hybrid between *A.cepa* and *A.fistulosum* Linnaeus, 1753) (Singh et al. 1967; Langer and Koul 1983; Puizina and Papea 1996) show variable number of satellited chromosomes (Bozzini 1964; Singh et al. 1967; Koul and Gohil 1971; Langer and Koul 1983; Puizina and Papea 1996). Many of the conventional staining and C-banding studies showed the presence of two satellite chromosomes in *A.cepa* (Ved Brat and Dhingra 1973; Fiskesjo 1975; Bhattacharyya 1976; Talukder and Sen 2000). Application of differential staining with sequence specific fluorochromes elucidated two NORs in *A.cepa* (Kim et al. 2002). However, reports claiming variable numbers of NORs (Battaglia 1957; Sato 1981; Puizina and Papea 1996) could not be ruled out. With the application of silver staining, 1–4 active NORs in the satellite region were observed (Sato 1981) while variable number of NORs (2–5) was elucidated by 45S-rDNA hybridization (Table 2). The 45S rDNA sites are distally located and found to co-occur with telomeric tandem repeats (18S). The 5S rDNA loci are reported to range from 2–4 and do not co-occur with the 45S rDNA site.

**Figure 2.**
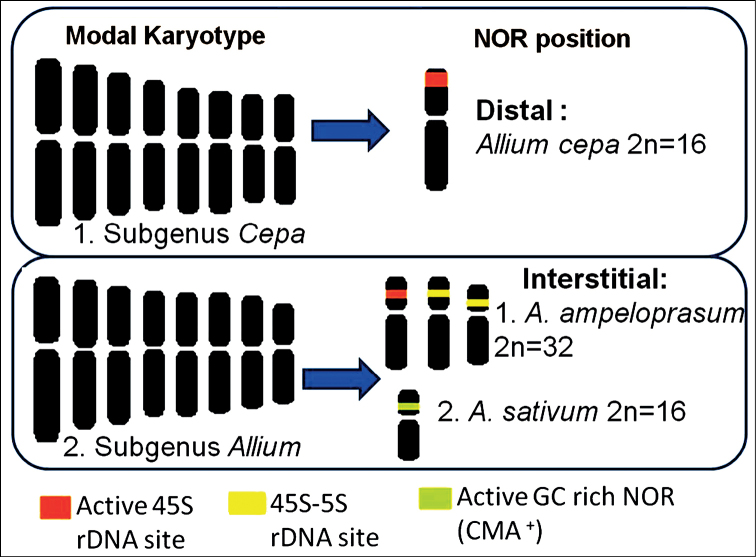
Diagram showing NOR landmarks based on globally published reports in the three species of the genus *Allium* occurring in India. The modal karyotypes for subgenera are adopted and modified after Peruzzi et al. (2017). Diagrams showing NORs are modified after the published reports on *A.ampeloprasum* (as *A.porrum* in Maragheh et al. 2019), *A.sativum* (Bacelar et al. 2021) and *A.cepa* (Fajkus et al. 2016).

One interesting feature is that satellites occur mostly in the short arms except for some cases in the subgenera *Allium* and *Amerallium* (Peruzzi et al. 2017). The same phenomenon has been found to exist in case of *A.cepa*, *A.sativum* and *A.ampeloprasum* (Kim et al. 2002; Maragheh et al. 2019; Bacelar et al. 2021). However, the localization of satellites in the species of *Amerallium* and other subgenera of Indian occurrence opens interesting scope of future study. The major difference between subgenera *Allium* and *Cepa* lie in the localization of the NORs rather than numbers of rDNA loci. The NORs are interstitial in *Allium* and distal in *Cepa* (Fig. 2) as confirmed by heterochromatic CMA banding, Ag-NOR staining as well as rDNA FISH (Kim et al. 2002; Fajkus et al. 2016; Maragheh et al. 2019; Bacelar et al. 2021).

## ﻿Chromosome specialization in *A.cepa*

Telomeres and rDNA loci are the two especially variable features of *A.cepa* chromosomes. Many authors have previously argued that genomic rearrangements are responsible for positional variations of 45S rDNA loci in *A.cepa* (Ricroch et al. 1992; Do et al. 2001; Mancia et al. 2015). The rDNA sequences have been found to contain Copia-like retroelements in *A.cernuum* Roth, 1798 that were dispersed via homogenization mechanisms (Fajkus et al. 2016). The rDNA loci in *A.cepa* have been observed to co-occur with telomeric repeats although telomeres evolved independently of rDNA sequences (Fajkus et al. 2016).

The plant telomere was once thought to be composed of *Arabidopsis* Heynhold, 1842 prototype TTTAGGG repeats (Richards and Ausubel 1988). Exception to this was observed in Asparagales, where an 80 million years old mutation gave rise to human type (TTAGGG) repeat in the family Iridaceae (Adams et al. 2001; Weiss and Scherthan 2002; Sýkorová et al. 2003) and subfamily Allioideae (Sýkorová et al. 2006). The genus *Allium* is different from all other subfamilies of Amaryllidaceae and also other plant groups in terms of the unique telomere sequence. The telomeric sequence (TTATGGGCTCGG)_n_ surfaced long back (Fuchs et al. 1995) and is neither *Arabidopsis* nor human type. The sequence has been found to be conserved in *Allium*, probing for monophyletic origin of this genus (Fajkus et al. 2016). The telomeres of land plants, including the unique ones like that of Amaryllids, have received less attention (Peska and Garcia 2020). For example, telomeric repeat in *Arabidopsisthaliana* (Linnaeus, 1753) Heynhold, 1842 is a Pol III transcribed lncRNA (Fajkus et al. 2019). Hence, the *Allium* and non-*Allium* taxa of Amaryllidaceae provide excellent scope for studying telomere evolution in eukaryotes.

## ﻿Recent updates on cytogenetic relationships

A robust phylogenetic analysis supported by genome size and karyotype parameters was found to elucidate the evolution of Gilliesieae of Allioideae (Pellicer et al. 2017). The phylogenetic background of the genus *Allium* has paved way for refinement of classification, inter-species relationships and cytogeographic evolution (Friesen et al. 2006; Gurushidze et al. 2007, 2008; Fritsch et al. 2010; Li et al. 2010, 2017; Abugalieva et al. 2017; Herden et al. 2016; Huo et al. 2019; Costa et al. 2020). Global sampling of 207 species of *Allium* (Allieae) highlighted the ancestral number (x=8) and the reasons behind symmetric karyotype evolution (Peruzzi et al. 2017).

The utility of cytogenetic mapping remains unparallel to investigate synteny comparison between phylogenetically related species that has been employed to interpret chromosome evolution in *Allium* crop species from Russia (Khrustaleva et al. 2019). The presence of flavonoids and sulphur-containing compounds are responsible for the onion’s characteristic flavour and the enzyme alliinase is part of the biosynthesis (Lancaster and Collin 1981). Recent techniques like ultra-sensitive tyramide-FISH (tyr-FISH) and SteamDrop protocol have facilitated the physical detection of the alliinase as well as chalcone synthase genes along with expressed sequence tag (EST) markers. The bulb alliinase gene was located on the long arm of chromosome 4 in *A.cepa* and *A.schoenoprasum* while the same gene was found in the short arm of chromosome 4 in the related (*A.fistulosum*, *A.altaicum* Pallas, 1773, *A.oschaninii* O. Fedtschenko, 1906, and *A.pskemense* B. Fedtschenko, 1905) and phylogenetically distant species (*A.roylei* and *A.nutans* Linnaeus, 1753) (Khrustaleva et al. 2019). Khrustaleva et al. (2019) proposed a pericentric inversion model for rearrangements in chromosome 4 in line with divergence of *A.cepa* and *A.fistulosum*, responsible for breaking collinearity of the genes controlling flavour and bulb colour. This particular report focussed on genomic kinship and genomic rearrangement among the closely related *Allium* species. Also, the practical benefit of molecular cytogenetic mapping becomes apparent in terms of suitably utilizing the genomic resources for onion breeding. These studies would also help to address genomic relationships among *A.cepa*, *A.schoenoprasum* and *A.roylei*, occurring in India.

## ﻿Summary and future prospects

Considering the impact of cytogenetic investigation in *Allium* phylogeny at a global scale, it is unfortunate to notice the lack of attention in an Indian context in spite of species abundance. Although *A.cepa* has often been regarded as the common material for cytogenetic analysis and the popular ‘*Alliumcepa* test’ (Pathiratne et al. 2015; Bonciu et al. 2018), systematic chromosome analysis is still missing in Indian *A.cepa* as well as other species. The present dataset and existing references are not exhaustive but furnish the prerequisite to search for further chromosomal landmarks (NORs, genome size etc) and complement future phylogenetic studies or cyto-geographical evolution of *Allium*, involving the unexplored wild and endemic species in the subcontinent. The crops, onion and garlic, have been admired from ancient time in global cuisines and Indian culinary practices (c.a. 5000 years ago) and continue to be tremendously important in agriculture and pharmaceutical industries (Rana et al. 2011; Nile and Park 2013). The cultivation of onions is challenged by a number of biotic threats which are the direct or indirect manifestation of the current climatic adversity (Le et al. 2021). Identification of wild relatives of the crop having high resistance is germane to address available genomic sources (Dempewolf et al. 2014), which is necessary for *Allium* crop species of India (Gedam et al. 2021). Interesting discoveries on the ‘neodomesticate’ western Himalaya taxon *A.negianum* (Pandey et al. 2021) along with other endemic less-known species (*A.stracheyi*, *A.roylei*, *A.wallichii* and *A.przewaliskianum*) are assets of Indian repository in line with global assemblages. The genomic attributes of Indian *Allium* germplasm as outlined in this review, could help strategic upgradation of cultivation practices.

## ﻿Author contribution

Conceptualization, supervision, project administration and funding: SJ, MML, DO, SRR, SRY, MKD, SNR, RCV. Data Curation and data analysis: BKB, SS, DRC, SDP. Writing and Editing: BKB, SS, DRC, MML, DO, SJ.

## ﻿ORCID

**Biplab Kumar Bhowmick** https://orcid.org/0000-0001-6029-1098

**Sayantika Sarkar** https://orcid.org/0000-0002-8738-9500

**Dipasree Roychowdhury** https://orcid.org/0000-0001-7537-4056

**Sayali D. Patil** https://orcid.org/0000-0000-0000-00000

**Manoj M. Lekhak** https://orcid.org/0000-0001-5753-2225

**Deepak Ohri** https://orcid.org/0000-0001-6327-4330

**Satyawada Rama Rao** https://orcid.org/0000-0003-0309-720X

**S. R. Yadav** https://orcid.org/0000-0001-6728-5483

**R. C. Verma** https://orcid.org/0000-0000-0000-00000

**Manoj K. Dhar** https://orcid.org/0000-0002-8777-6244

**S. N. Raina** https://orcid.org/0000-0002-4916-3359

**Sumita Jha** https://orcid.org/0000-0002-1375-2768

## References

[B1] Abdali S Miri SM (2020) Chromosome counts for six species of *Allium* (Amaryllidaceae) from Iran. The Iranian Journal of Botany 26(2): 179–187.

[B2] Abugalieva S Volkova L Genievskaya Y Ivaschenko A Kotukhov Y Sakauova G Turuspekov Y (2017) Taxonomic assessment of *Allium* species from Kazakhstan based on ITS and *matK* markers. BMC Plant Biology 17(2): 51–60. 10.1186/s12870-017-1194-0 29297332 PMC5751797

[B3] Adams SP Leitch IJ Bennett MD Leitch AR (2001) *Aloe* L. – a second plant family without (TTTAGGG) telomeres. Chromosoma 109: 201–205. 10.1007/s004120050429 10929199

[B4] Ahirwar R Verma RC (2015) Colchicine induced asynaptic chromosomal behavior at meiosis in *Alliumcepa* L. The Nucleus 58: 47–51. 10.1007/s13237-015-0133-4

[B5] Anderson LK Stack SM Fox MH Chuanshan Z (1985) The relationship between genome size and synaptonemal complex length in higher plants. Experimental Cell Research 156(2): 367–378. 10.1016/0014-4827(85)90544-0 3967684

[B6] Angiosperm Phylogeny Group (2016) An update of the Angiosperm Phylogeny Group classification for the orders and families of flowering plants: APG IV. Botanical Journal of the Linnean Society 181: 1–20. 10.1111/boj.12385

[B7] Ao C (2008) Chromosome numbers and karyotypes of *Alliumprzewalskianum* populations. Acta Biologica Cracoviensia Series Botanica 50: 43–49.

[B8] Arumuganathan K Earle ED (1991) Estimation of nuclear DNA content of plants by flow cytometry. Plant Molecular Biology Reporter 9: 229–241. 10.1007/BF02672073

[B9] Bacelar PAA Feitoza LL Valente SES Gomes RLF Martins LV Almeida PM Silva VB Lopes ACA Carvalh R Peron AP (2021) Variations in heterochromatin content reveal important polymorphisms for studies of genetic improvement in garlic (*Alliumsativum* L.). Brazilian Journal of Biology 83: e243514. 10.1590/1519-6984.243514 34133490

[B10] Baranyi M Greilhuber J (1999) Genome size in *Allium*: in quest of reproducible data. Annals of Botany 83(6): 687–695. 10.1006/anbo.1999.0871

[B11] Bartolo G Brullo S Pavone P Terrasi MC (1984) Cytotaxonomical notes on some Liliaceae of N. Cyrenaica. Webbia 38: 601–622. 10.1080/00837792.1984.10670329

[B12] Battaglia E (1957) *Alliumascalonicum* L., *A.fistulosum* L., *A.cepa* L.: Analisi Cariotipica. Caryologia 10: 1–28. 10.1080/00087114.1957.10797610

[B13] Bhattacharyya R (1976) A tetraploid *Alliumcepa* from Bangladesh. Cytologia 41: 513–521. 10.1508/cytologia.41.513

[B14] Bhowmick BK Jha S (2022) A critical review on cytogenetics of Cucurbitaceae with updates on Indian taxa. Comparative Cytogenetics 16: 93–125. 10.3897/compcytogen.v16.i2.79033 36761811 PMC9849056

[B15] Bonciu E Firbas P Fontanetti CS Wusheng J Karaismailoğlu MC Liu D Menicucci F Pesnya DS Popescu A Romanovsky AV Schiff S (2018) An evaluation for the standardization of the *Alliumcepa* test as cytotoxicity and genotoxicity assay. Caryologia 71(3): 191–209. 10.1080/00087114.2018.1503496

[B16] Borowska-Zuchowska N Senderowicz M Trunova D Kolano B (2022) Tracing the evolution of the angiosperm genome from the cytogenetic point of view. Plants 11(6): e784. 10.3390/plants11060784 PMC8953110 35336666

[B17] Bozzini A (1964) On the karyotype of a viviparous onion, known as AlliumcepaL.varviviparum (Metzg.) Alef. Caryologia 17: 459–470. 10.1080/00087114.1964.10796142

[B18] Cai Q Chinnappa CC (1987) Giemsa C-banded karyotypes of seven North American species of *Allium*. American Journal of Botany 74: 1087–1092. 10.2307/2443949

[B19] Carta A Bedini G Peruzzi L (2020) A deep dive into the ancestral chromosome number and genome size of flowering plants. New Phytologist 228(3): 1097–1106. 10.1111/nph.16668 32421860

[B20] Chakravarty B Sen S (1992) DNA and protein contents in different varieties of *Alliumcepa* and *Alliumsativum*. Allium Improvement Newsletter 1: 61–66.

[B21] Chunying X Jiemei X Jianquan L (2000) Karyotype studies of *Alliumprattii* among 4 populations in southern Qinghai. Acta Botanica Boreali-Occidentalia Sinica 20(2): 288–293.

[B22] Cortes F Escalza P (1986) Analysis of different banding patterns and late replicating regions in chromosomes of *Alliumcepa*, *A.sativum* and *A.nigrum*. Genetica 71: 39–46. 10.1007/BF00123231

[B23] Cortes F Gonzalez-Gil G Hazen MJ (1983) C-banding and sister chromatid exchanges in three species of the genus *Allium* (*A.cepa*, *A.ascalonicum* and *A.sativum*). Caryologia 36: 203–210. 10.1080/00087114.1983.10797661

[B24] Costa L Jimenez H Carvalho R Carvalho-Sobrinho J Escobar I Souza G (2020) Divide to conquer: evolutionary history of Allioideae tribes (Amaryllidaceae) is linked to distinct trends of karyotype evolution. Frontiers in Plant Science 11: e320. 10.3389/fpls.2020.00320 PMC7155398 32318079

[B25] Darlington CD Haque A (1955) The timing of mitosis and meiosis in *Alliumascalonicum*: A problem of differentiation. Heredity 9: 117–127. 10.1038/hdy.1955.6

[B26] Darlington CD Wylie AP (1955) Chromosome Atlas of Flowering Plants. George Allen & Unwin Ltd., London.

[B27] Dempewolf H Eastwood RJ Guarino L Khoury CK Muller JV Toll J (2014) Adapting agriculture to climate change: a global initiative to collect, conserve, and use crop wild relatives. Agroecology and Sustainable Food Systems 38(4): 369–377. 10.1080/21683565.2013.870629

[B28] dGRIP (2022) Database for genome related information in Indian plants. http://indianpcd.com/

[B29] Do GS Seo BB (2000) Phylogenetic relationships among Alliumsubg.Rhizirideum species based on the molecular variation of 5S rRNA genes. Korean Journal of Biological Sciences 4(1): 77–85. 10.1080/12265071.2000.9647527

[B30] Do GS Seo BB Yamamoto M Suzuki G Mukai Y (2001) Identification and chromosomal location of tandemly repeated DNA sequences in *Alliumcepa*. Genes & Genetic Systems 76: 53–60. 10.1266/ggs.76.53 11376552

[B31] Dutta M Bandyopadhyay M (2014) Comparative karyomorphological studies of three edible locally important species of *Allium* from India. The Nucleus 57(1): 25–31. 10.1007/s13237-014-0106-z

[B32] Dutta M Negi KS Bandyopadhyay M (2015) Novel cytogenetic resources of wild *Allium* (Amaryllidaceae) from India. The Nucleus 58(1): 15–21. 10.1007/s13237-015-0130-7

[B33] El-Gadi A Elkington TT (1977) Numerical taxonomic studies on species in AlliumsubgenusRhizirideum. New Phytologist 79: 183–201. 10.1111/j.1469-8137.1977.tb02195.x

[B34] Fajkus P Peška V Sitová Z Fulnečková J Dvořáčková M Gogela R Sýkorová E Hapala J Fajkus J (2016) *Allium* telomeres unmasked: the unusual telomeric sequence (CTCGGTTATGGG)*n* is synthesized by telomerase. The Plant Journal 85(3): 337–347. 10.1111/tpj.13115 26716914

[B35] Fajkus P Peška V Závodník M Fojtová M Fulnečková J Dobias Š Kilar A Dvořáčková M Zachová D Nečasová I Sims J Sýkorová E Fajkus J (2019) Telomerase RNAs in land plants. Nucleic Acids Research 47(18): 9842–9856. 10.1093/nar/gkz695 31392988 PMC6765143

[B36] Fiskesjo G (1975) Chromosomal relationships between three species of *Allium* as revealed by C-banding. Hereditas 81: 23–32. 10.1111/j.1601-5223.1975.tb01010.x

[B37] Friesen N Fritsch RM Blattner FR (2006) Phylogeny and new intrageneric classification of *Allium* (Alliaceae) based on nuclear ribosomal DNA ITS sequences. Aliso: A Journal of Systematic and Floristic Botany 22(1): 372–395. 10.5642/aliso.20062201.31

[B38] Friesen NV (1985) Chromosome numbers in the representatives of the family Alliaceae from Siberia. Botanicheskii Zhurnal SSSR 70(7): 1001–1002.

[B39] Friesen NV (1986) Chromosome numbers of the representatives of the family Alliaceae from Siberia. Botanicheskii Zhurnal 71: 113–115.

[B40] Fritsch RM Blattner FR Gurushidz M (2010) New classification of AlliumL.subg.Melanocrommyum (Webb & Berthel.) Rouy (Alliaceae) based on molecular and morphological characters. Phyton (Horn) 49(2): 145–220.

[B41] Fu J Zhang H Guo F Ma L Wu J Yue M Zheng X Qiu Z Li L (2019) Identification and characterization of abundant repetitive sequences in *Alliumcepa*. Scientific Reports 9: e16756. 10.1038/s41598-019-52995-9 PMC6856378 31727905

[B42] Fuchs J Brandes A Schubert I (1995) Telomere sequence localization and karyotype evolution in higher plants. Plant Systematics and Evolution 196: 227–241. 10.1007/BF00982962

[B43] Gedam PA Thangasamy A Shirsat DV Ghosh S Bhagat KP Sogam OA Gupta AJ Mahajan V Soumia PS Salunkhe VN Khade YP Gawande SJ Hanjagi PS Shiv Ramakrishnan R Singh M (2021) Screening of onion (*Alliumcepa* L.) genotypes for drought tolerance using physiological and yield based indices through multivariate analysis. Frontiers in Plant Science 12: e122. 10.3389/fpls.2021.600371 PMC7900547 33633759

[B44] Ghosh S Roy SC (1977) Orientation of interphase chromosomes as detected by Giemsa C-bands. Chromosoma 61: 49–55. 10.1007/BF00292679 885027

[B45] Gohil RN Kaul R (1979) Seed progeny studies in *Alliums*. I. Numerical variants in the progeny of tetraploid *Alliumtuberosum* Rottl. ex Spreng. Beiträge zur Biologie der Pflanzen 54: 304–309.

[B46] Gohil RN Kaul R (1980a) Studies on male and female meiosis in Indian *Allium* I. Four diploid species. Chromosoma 77: 123–127. 10.1007/BF00329538

[B47] Gohil RN Kaul R (1980b) An interesting variation in the development of the female gametophyte of *Alliumconsanguineum*. Caryologia 33(2): 295–297. 10.1080/00087114.1980.10796843

[B48] Gohil RN Kaul R (1981) Studies on male and female meiosis in Indian *Allium* II, Autotetraploid *Alliumtuberosum*. Chromosoma 82: 735–739. 10.1007/BF00285778

[B49] Gohil RN Koul AK (1971) Desynapsis in some diploid and polyploidy species of *Allium*. Canadian Journal of Genetics and Cytology 13: 723–728. 10.1139/g71-104

[B50] Gohil RN Koul AK (1973) Some adaptive genetic-evolutionary processes accompanying polyploidy in the Indian *Alliums*. Botaniska Notiser 126: 426–432.

[B51] Gohil RN Koul AK (1977) The cause of multivalent suppression in *Alliumampeloprasum* L. Beiträge zur Biologie der Pflanzen 53: 473–478.

[B52] Gohil RN Koul AK (1981) Cytology of the tetraploid *Alliumchinense* G. Don. Caryologia 34(1): 73–81. 10.1080/00087114.1981.10796874

[B53] Gohil RN Koul AK (1983) Seed progeny studies in *Alliums*. II. Male meiosis in the progeny plants of tetraploid *Alliumtuberosum* Rottl. ex Spreng. Cytologia 48: 109–118. 10.1508/cytologia.48.109

[B54] Goldblatt P Lowry PP (2011) The Index to Plant Chromosome Numbers (IPCN): three decades of publication by the Missouri Botanical Garden come to an end. Annals of the Missouri Botanical Garden 98(2): 226–227. 10.3417/2011027

[B55] Gu Z Wang L Sun H Wu S (1993) A cytological study of some plants from Qinghai-Xizang Plateau. Acta Botanica Yunnanica 15: 377–384.

[B56] Gurushidze M Fritsch RM Blattner FR (2008) Phylogenetic analysis of Alliumsubg.Melanocrommyum infers cryptic species and demands a new sectional classification. Molecular Phylogenetics and Evolution 49(3): 997–1007. 10.1016/j.ympev.2008.09.003 18824112

[B57] Gurushidze M Fuchs J Blattner FR (2012) The evolution of genome size variation in drumstick onions (AlliumsubgenusMelanocrommyum). Systematic Botany 37(1): 96–104. 10.1600/036364412X616675

[B58] Gurushidze M Mashayekhi S Blattner FR Friesen N Fritsch RM (2007) Phylogenetic relationships of wild and cultivated species of AlliumsectionCepa inferred by nuclear rDNA ITS sequence analysis. Plant Systematics and Evolution 269(3): 259–269. 10.1007/s00606-007-0596-0

[B59] Halkka L (1985) Chromosome counts of Finnish vascular plants. Annales Botanici Fennici 22: 315–317.

[B60] Haston E Richardson JE Stevens PF Chase MW Harris DJ (2009) The Linear Angiosperm Phylogeny Group (LAPG) III: a linear sequence of the families in APG III. Botanical Journal of the Linnean Society 161(2): 128–131. 10.1111/j.1095-8339.2009.01000.x

[B61] Herden T Hanelt P Friesen N (2016) Phylogeny of AlliumL.subgenusAnguinum (G. Don. ex WDJ Koch) N. Friesen (Amaryllidaceae). Molecular Phylogenetics and Evolution 95: 79–93. 10.1016/j.ympev.2015.11.004 26639102

[B62] Huang R Wei R Yan Y (1985) Discovery of spontaneous triploid of *Alliumtuberosum*. Journal of Wuhan Botanical Research 3: 429–431.

[B63] Huang R Xu J Hong Y (1995) A study on karyotypes and their evolutionary trends in Alliumsect.Bromatorrhiza Ekberg (Liliaceae). Cathaya 7: 133–145.

[B64] Huo Y Gao L Liu B Yang Y Kong S Sun Y Yang Y Wu X (2019) Complete chloroplast genome sequences of four *Allium* species: comparative and phylogenetic analyses. Scientific Reports 9(1): 1–14. 10.1038/s41598-019-48708-x 31439882 PMC6706373

[B65] Islam-Faridi N Sakhnokho HF Nelson CD (2020) New chromosome number and cyto-molecular characterization of the African Baobab (*Adansoniadigitata* L.) – “The Tree of Life”. Scientific Reports 10: e13174. 10.1038/s41598-020-68697-6 PMC7413363 32764541

[B66] Jha S Raina SN Ohri D Verma RC Dhar MK Lekhak MM Yadav SR Mahadev N Satyawada RR (2019) A new online database on genome-related information of Indian plants. Plant Systematics and Evolution 305(9): 837–843. 10.1007/s00606-019-01602-5

[B67] Johnson MAT Zhatay N (1996) Cytology of Alliumsect.Allium. In: Mathew B (Ed.) A Review of Allium sect.*Allium*. Kew Royal Botanic Gardens, Kew, 17–31.

[B68] Jones RN Rees H (1968) Nuclear DNA variation in *Allium*. Heredity 23: 591–605. 10.1038/hdy.1968.76

[B69] Joshi CP Ranjekar PK (1982) Visualization and distribution of heterochromatin in interphase nuclei of several plant species as revealed by a new giemsa banding technique. Cytologia 47: 471–480. 10.1508/cytologia.47.471

[B70] Katayama Y (1928) The chromosome number in *Phaseolus* and *Allium* and observation on the size of stomata in different species of *Triticum*. Jour. Sci. Agric. Soc. Tokyo 303: 52–54.

[B71] Khazanehdari KA Jones GH (1996) Meiotic synapsis of the *Alliumporrum* B chromosome: evidence for a derived isochromosome origin. Genome 39(6): 1199–1204. 10.1139/g96-151 18469966

[B72] Khoshoo TN Sharma VB (1959) Cytology of the autotriploid *Alliumrubellum*. Chromosoma 10: 136–143. 10.1007/BF00396567 13652349

[B73] Khrustaleva L Kudryavtseva N Romanov D Ermolaev A Kirov I (2019) Comparative Tyramide-FISH mapping of the genes controlling flavor and bulb color in *Allium* species revealed an altered gene order. Scientific Reports 9(1): 1–11. 10.1038/s41598-019-48564-9 31427665 PMC6700127

[B74] Kim ES Punina EO Rodionov AV (2002) Chromosome CPD (PI/DAPI) and CMA/DAPI-banding patterns in *Alliumcepa* L. Russian Journal of Genetics 38(4): 489–496. 10.1023/A:1015250219322 12018166

[B75] Kohli B Gohil RN (2011) Is *Alliumroylei* Stearn still evolving through multiple interchanges? The Nucleus 54(1): 19–23. 10.1007/s13237-011-0018-0

[B76] Kohli B Kaul V (2013) Sterility in *Alliumroylei* Stearn – A lesser explored taxon. International Journal of Pharma and Bio Sciences 4(1): 741–746.

[B77] Kojima A Hinata K Noda S (1991) An improvement of squash method for cytological study of female meiosis in *Alliumtuberosum*, Liliaceae. Chromosome Information Service (CIS) 50: 5–7.

[B78] Koul AK (1963) A spontaneously occurring reciprocal translocation heterozygote of *Alliumcepa*. The Journal of Indian Botanical Society 42: 416–419.

[B79] Koul AK (1966) Structural hybridity in *Alliumatropurpureum* Waldst. & Kit. Journal of Cytology and Genetics 1: 87–89.

[B80] Koul AK Gohil RN (1970a) Causes averting sexual reproduction in *Alliumsativum* L. Cytologia 35: 197–202. 10.1508/cytologia.35.197

[B81] Koul AK Gohil RN (1970b) Cytology of tetraploid *Alliumampeloprasum* with chiasma localization. Chromosoma 29: 12–19. 10.1007/BF01183658

[B82] Koul AK Gohil RN (1971) Further studies on natural triploidy in viviparous onion. Cytologia 36(2): 253–261. 10.1508/cytologia.36.253

[B83] Koul AK Sharma MC Gohil RN (1971) Cytology of the tetraploid *Alliumrubellum* Bieb. Caryologia 24: 149–155. 10.1080/00087114.1971.10796422

[B84] Kumar S Thonger T (2018) Karyomorphology of five *Allium* species from Nagaland, North-Eastern Region of India. Jordan Journal of Biological Sciences 11(1): 9–15.

[B85] Kumari K Saggoo MIS (2016) Male meiosis and morphometric analysis of ethnobotanically important *Alliumcarolinianum* DC. from Kinnaur district of Himachal Pradesh, India. Asian Journal of Pharmaceutical and Clinical Research 9(4): 396–398.

[B86] Kurosawa S (1966) Cytological studies on some eastern Himalayan plants. In: Hara H (Ed.) The Flora of Eastern Himalaya.University of Tokyo, Japan, 658–690.

[B87] Kurosawa S (1979) Notes on chromosome numbers of Spermatophytes II. Journal of Japanese Botany 54: 155–160.

[B88] Labani RM Elkington TT (1987) Nuclear DNA variation in the genus *Allium* L. (Liliaceae). Heredity 59: 119–128. 10.1038/hdy.1987.103

[B89] Lancaster JE Collin HA (1981) Presence of alliinase in isolated vacuoles and of alkyl cysteine sulphoxides in the cytoplasm of bulbs of onion (*Alliumcepa*). Plant Science Letters 22(2): 169–176. 10.1016/0304-4211(81)90139-5

[B90] Langer A Koul AK (1983) Studies on nucleolus and nucleolar chromosomes in angiosperms VII. Nature of nucleolar chromosome polymorphism in Alliumcepavar.viviparum (Metzg.) Alef. Cytologia 48: 323–332. 10.1508/cytologia.48.323

[B91] Le D Audenaert K Haesaert G (2021) *Fusarium* basal rot: profile of an increasingly important disease in *Allium* spp. Tropical Plant Pathology 46: 241–253. 10.1007/s40858-021-00421-9

[B92] Lee SH Do GS Seo BB (1999) Chromosomal localization of 5S rRNA gene loci and the implications for relationships within the *Allium* complex. Chromosome Research 7: 89–93. 10.1023/a:1009222411001 10328620

[B93] Levan A (1934) Cytological studies in *Allium*, V *Alliummacranthum*. Hereditas 18: 349–359. 10.1111/j.1601-5223.1934.tb02619.x

[B94] Levan A (1940) Meiosis of *Alliumporrum*. A tetraploid species with chiasma localisation. Hereditas 26: 454–462. 10.1111/j.1601-5223.1940.tb03248.x

[B95] Li MJ Guo XL Li J Zhou SD Liu Q He XJ (2017) Cytotaxonomy of *Allium* (Amaryllidaceae) subgenera *Cyathophora* and Ameralliumsect.Bromatorrhiza. Phytotaxa 331(2): 185–198. 10.11646/phytotaxa.331.2.3

[B96] Li QQ Zhou SD He XJ Yu Y Zhang YC Wei XQ (2010) Phylogeny and biogeography of *Allium* (Amaryllidaceae: Allieae) based on nuclear ribosomal internal transcribed spacer and chloroplast rps16 sequences, focusing on the inclusion of species endemic to China. Annals of Botany 106: 709–733. 10.1093/aob/mcq177 20966186 PMC2958792

[B97] Li R Liu L Wang X (1985) Karyotype analysis on the different cultivars of *Alliumtuberosum* Rottle. Chinese Bulletin of Botany 3(5): 43–46.

[B98] Lu Y Deng Y Lu L He X (2017) Karyotypes of nineteen populations of four species in AlliumsubgenusAnguinum. Guangxi Zhiwu/Guihaia 37(7): 811–821.

[B99] Mancia FH Sohn SH Ahn YK Kim DS Kim JS Kwon YS Kim CW Lee TH Hwang YJ (2015) Distribution of various types of repetitive DNAs in *Alliumcepa* L. based on dual color FISH. Horticulture, Environment and Biotechnology 56(6): 793–799. 10.1007/s13580-015-1100-3

[B100] Maragheh FP Janus D Senderowicz M Haliloglu K Kolano B (2019) Karyotype analysis of eight cultivated *Allium* species. Journal of Applied Genetics 60(1): 1–11. 10.1007/s13353-018-0474-1 30353472 PMC6373409

[B101] Mehra PN Pandita TK (1979) IOPB chromosome number reports LXIV. Taxon 28: 391–408. 10.1002/j.1996-8175.1979.tb00530.x

[B102] Mehra PN Sachdeva SK (1975) IOPB chromosome number reports XLIX. Taxon 24: 501–516. 10.1002/j.1996-8175.1975.tb00341.x

[B103] Mehra PN Sachdeva SK (1976) Cytological observations on some West Himalayan monocots. III. Alliaceae. Cytologia 41: 23–30. 10.1508/cytologia.41.23

[B104] Murin A (1976) Polyploidy and mitotic cycle. The Nucleus 19: 192–195.

[B105] Nanda S Chand SK Mandal P Tripathy P Joshi RK (2016) Identification of novel source of resistance and differential response of *Allium* genotypes to purple blotch pathogen, *Alternariaporri* (Ellis) Ciferri. The Plant Pathology Journal 32(6): 519–527. 10.5423/PPJ.OA.02.2016.0034 27904458 PMC5117860

[B106] Nanushyan ER Polyakov VJ (1989) Zavisimost mezhdu kolichestvom DNK, tolshchinoy mitoticheskikh khromosom i obyemom pyltsevykh zeren u nekotorykh vidov roda *Allium* L. Biologicheskiye Nauki (Moskva) 8: 50–56.

[B107] Narayan RKJ (1988) Constraints upon the organization and evolution of chromosomes in *Allium*. Theoretical and Applied Genetics 75: 319–329. 10.1007/BF00303971

[B108] Nath S Sarkar S Patil SD Saha PS Lekhak MM Ray S Rama Rao S Yadav SR Verma RC Dhar MK Raina SN Jha S (2022) Cytogenetic diversity in Scilloideae (Asparagaceae): a comprehensive recollection and exploration of karyo-evolutionary trends. The Botanical Review. 10.1007/s12229-022-09279-1

[B109] Nile SH Park SW (2013) Total phenolics, antioxidant and xanthine oxidase inhibitory activity of three colored onions (*Alliumcepa* L.). Frontiers in Life Science 7(3–4): 224–228. 10.1080/21553769.2014.901926

[B110] Ogura H Kondo K Morimoto M Aizawa T Chen Z Hong D (1999) A karyological study of *Alliumgrayi* Regel and *A.chinense* G. Don in Sichuan Province, China. Chromosome Science 3: 119–122.

[B111] Ohri D Fritsch RM Hanelt P (1998) Evolution of genome size in *Allium* (Alliaceae). Plant Systematics and Evolution 210: 57–86. 10.1007/BF00984728

[B112] Ohri D Pistrick K (2001) Phenology and genome size variation in *Allium* L.- a tight correlation? Plant Biology (Stuttgart) 3: 654–660. 10.1055/s-2001-19362

[B113] Ohri M (1990) Studies on the factor of existence in *Alliumchinense* guessed from elimination of the constitution of chromosomes. Journal of the Faculty of Agriculture, Shinshu University 27: 49–90.

[B114] Olszewska MJ Osiecka R (1982) The relationship between 2C DNA content, life cycle type, systematic position, and the level of DNA endoreplication in nuclei of parenchyma cells during growth and differentiation of roots in some monocotyledonous species. Biochemie und Physiologie der Pflanzen 177(4–5): 319–336. 10.1016/S0015-3796(82)80026-7

[B115] Oyuntsetseg B Friesen N Darikhand D (2013) *Alliumcarolinianum* DC., A new species to the outer Mongolia. Turczaninowia 16(2): 88–90.

[B116] Pandey A Malav PK Rai KM Ahlawat SP (2022) Genus *Allium* L. of the Indian Region: A field guide for germplasm collection and identification. ICAR-National Bureau of Plant Genetic Resources, New Delhi.

[B117] Pandey A Malav PK Rai MK Ahlawat SP (2021) ‘Neodomesticates’ of the Himalayan *Allium* spices (*Allium* species) in Uttarakhand, India and studies on eco-geography and morphology. Genetic Resources and Crop Evolution 68(5): 2167–2179. 10.1007/s10722-021-01164-x

[B118] Pandita TK Mehra PN (1981a) Cytology of *Alliums* of Kashmir Himalayas, III. Male Meiosis. The Nucleus 24(3): 147–151.

[B119] Pandita TK Mehra PN (1981b) Cytology of *Alliums* of Kashmir Himalayas, I. Wild species. The Nucleus 24(1, 2): 5–10.

[B120] Pathiratne A Hemachandra CK De Silva N (2015) Efficacy of *Alliumcepa* test system for screening cytotoxicity and genotoxicity of industrial effluents originated from different industrial activities. Environmental Monitoring and Assessment 187(12): 1–12. 10.1007/s10661-015-4954-z 26547320

[B121] Pedersen K Wendelbo P (1966) Chromosome numbers of some SW Asian *Allium* species. Blyttia 24: 307–313.

[B122] Pellicer J Hidalgo O Walker J Chase MW Christenhusz MJ Shackelford G Leitch IJ Fay MF (2017) Genome size dynamics in tribe Gilliesieae (Amaryllidaceae, subfamily Allioideae) in the context of polyploidy and unusual incidence of Robertsonian translocations. Botanical Journal of the Linnean Society 184(1): 16–31. 10.1093/botlinnean/box016

[B123] Pellicer J Leitch IJ (2020) The Plant DNA C-values database (release 7.1): an updated online repository of plant genome size data for comparative studies. New Phytologist 226(2): 301–305. 10.1111/nph.16261 31608445

[B124] Peruzzi L Carta A Altinordu F (2017) Chromosome diversity and evolution in *Allium* (Allioideae, Amaryllidaceae). Plant Biosystems – An International Journal Dealing with all Aspects of Plant Biology 151(2): 212–220. 10.1080/11263504.2016.1149123

[B125] Peska V Garcia S (2020) Origin, diversity, and evolution of telomere sequences in plants. Frontiers in Plant Science 11: e117. 10.3389/fpls.2020.00117 PMC7046594 32153618

[B126] Peska V Mandakova T Ihradska V Fajkus J (2019) Comparative dissection of three giant genomes: *Alliumcepa*, *Alliumsativum*, and *Alliumursinum*. International Journal of Molecular Sciences 20(3): e733. 10.3390/ijms20030733 PMC6387171 30744119

[B127] Phuong PTM Tashiro Y (2010) Study on diversity and chromosome numbers of edible *Allium* crops in Vietnam. Journal of Science and Development 8: 138–144.

[B128] Pogosian AI (1997) Chromosome numbers in some species of monocotyledons from the Transcaucasia. Botanicheskii Zhurnal (Moscow & Leningrad) 82(6): 117–118.

[B129] Puizina J Papea D (1996) Cytogenetical evidences for hybrid structure and origin of diploid and triploid shallots (Alliumcepavar.viviparum, Liliaceae) from Dalmatia (Croatia). Plant Systematics and Evolution 199: 203–215. 10.1007/BF00984905

[B130] Rana S Pal R Vaiphei K Sharma S Ola R (2011) Garlic in health and disease. Nutrition Research Reviews 24(1): 60–71. 10.1017/S0954422410000338 24725925

[B131] Ranjekar PK Pallotta D Lafontaine JG (1978) Analysis of plant genomes V. Comparative study of molecular properties of DNAs of seven *Allium* species. Biochemical Genetics 16: 957–970. 10.1007/BF00483747 743197

[B132] Rees H Narayan RKJ Hutchinson J (1979) DNA variation associated with the evolution of flowering species. The Nucleus 22: 1–5.

[B133] Rice A Glick L Abadi S Einhorn M Kopelman NM Salman-Minkov A Mayzel J Chay O Mayrose I (2015) The Chromosome Counts Database (CCDB) – a community resource of plant chromosome numbers. New Phytologist 206(1): 19–26. 10.1111/nph.13191 25423910

[B134] Richards EJ Ausubel FM (1988) Isolation of a higher eukaryotic telomere from *Arabidopsisthaliana*. Cell 53(1): 127–136. 10.1016/0092-8674(88)90494-1 3349525

[B135] Ricroch A Peffley EB Baker RJ (1992) Chromosomal location of rDNA in *Allium*: *in situ* hybridization using biotin-and fluorescein-labelled probe. Theoretical and Applied Genetics 83(4): 413–418. 10.1007/BF00226528 24202586

[B136] Ricroch A Yockteng R Brown SC Nadot S (2005) Evolution of genome size across some cultivated *Allium* species. Genome 48: 511–520. 10.1139/g05-017 16121247

[B137] Roy SC (1978) Polymorphism in giemsa banding patterns in *Alliumsativum*. Cytologia 43: 97–100. 10.1508/cytologia.43.97

[B138] Sato S (1981) Cytological studies on the satellited chromosomes of *Alliumcepa*. Caryologia 34(4): 431–440. 10.1080/00087114.1981.10796911

[B139] Sato S Kawamura S (1981) Cytological studies on the nucleolus and the NOR-carrying segments of *Alliumsativum*. Cytologia 46: 781–790. 10.1508/cytologia.46.781

[B140] Schubert I Wobus U (1985) In situ hybridization confirms jumping nucleolus organizing regions in *Allium*. Chromosoma 92: 143–148. 10.1007/BF00328466

[B141] Sen S (1973a) Structural hybridity intra- and interspecific level in Liliales. Folia Biologica (Cracow) 21: 183–197. 4733339

[B142] Sen S (1973b) Polysomaty and its significance in Liliales. Cytologia 38: 737–751. 10.1508/cytologia.38.737

[B143] Sen S (1974a) Cryptic structural changes in the evolution of cultivated *Alliums*. Indian Journal of Heredity 8: 41–50.

[B144] Sen S (1974b) Floral biology, meiosis, pollen cytology and cause of seed setting in *Alliumtuberosum* Rottl. Caryologia 27(1): 7–16. 10.1080/00087114.1974.10796557

[B145] Sen S (1974c) Nature and behaviour of B chromosomes in *Alliumstracheyii* Baker and *Urgineaindica* Kunth. Cytologia 39: 245–251. 10.1508/cytologia.39.245

[B146] Senderowicz M Nowak T Rojek-Jelonek M Bisaga M Papp L Weiss-Schneeweiss H Kolano B (2021) Descending dysploidy and bidirectional changes in genome size accompanied *Crepis* (Asteraceae) evolution. Genes 12(9): e1436. 10.3390/genes12091436 PMC8472258 34573417

[B147] Seo B (1977) Cytogenetic studies of some tetraploids in *Allium*. Korean Journal of Botany 20: 71–76.

[B148] Seo BB Kim JH (1975) Karyotypic analyses based on heterochromatin distribution in *Alliumfistulosum* and *Alliumascalonicum*. Korean Journal of Botany 18: 92–100.

[B149] Sharma AK Aiyangar HR (1961) Occurrence of B-chromosomes in diploid *Alliumstracheyi* Baker and their elimination in polyploids. Chromosoma 12(1): 310–317. 10.1007/BF00328926

[B150] Sharma G Gohil RN (2003) Cytology of *Alliumroylei* Stearn. 1. Meiosis in a population with complex interchanges. Cytologia 68: 115–119. 10.1508/cytologia.68.115

[B151] Sharma G Gohil RN (2004) Chromosomal chimeras in the male track of *Alliumtuberosum* Rottl. ex Spreng. Caryologia 57(2): 158–162. 10.1080/00087114.2004.10589386

[B152] Sharma G Gohil RN (2008) Intrapopulation karyotypic variability in *Alliumroylei* Stearn – a threatened species. Botanical Journal of the Linnean Society 158(2): 242–248. 10.1111/j.1095-8339.2008.00862.x

[B153] Sharma G Gohil RN (2011a) Occurrence of differential meiotic associations and additional chromosomes in the embryo-sac mother cells of *Alliumroylei* Stearn. Journal of Genetics 90: 45–49. 10.1007/s12041-011-0031-8 21677388

[B154] Sharma G Gohil RN (2011b) Occurrence of multivalents and additional chromosomes in the pollen mother cells of *Alliumcepa* L. The Nucleus 54(3): 137–140. 10.1007/s13237-011-0042-0

[B155] Sharma G Gohil RN (2013a) Origin and cytology of a novel cytotype of *Alliumtuberosum* Rottl. ex Spreng. (2n=48). Genetic Resources and Crop Evolution 60: 503–511. 10.1007/s10722-012-9852-4

[B156] Sharma G Gohil RN (2013b) Double hypoploid of *Alliumtuberosum* Rottl. ex Spreng. (2n=4x=30): its origin and cytology. Genetic Resources and Crop Evolution 60: 2283–2292. 10.1007/s10722-013-9995-y

[B157] Sharma G Gohil RN Kaul V (2011) Cytological status of *Alliumhookeri* Thwaites (2n=22). Genetic Resources and Crop Evolution 58: 1041–1050. 10.1007/s10722-010-9641-x

[B158] Shibata F Hizume M (2002) Evolution of 5S rDNA units and their chromosomal localization in *Alliumcepa* and *Alliumschoenoprasum* revealed by microdissection and FISH. Theoretical and Applied Genetics 105(2–3): 167–172. 10.1007/s00122-002-0950-0 12582516

[B159] Shopova M (1966) The nature and behaviour of supernumerary chromosomes in the *Rhizirideum* group of the genus *Allium*. Chromosoma 19: 149–158. 10.1007/BF00293680 5958570

[B160] Singh F Ved Brat S Khoshoo TN (1967) Natural triploidy in viviparous onions. Cytologia 32: 403–407. 10.1508/cytologia.32.403

[B161] Son JH Park KC Lee SI Jeon EJ Kim HH Kim NS (2012) Sequence variation and comparison of the 5S rRNA sequences in *Allium* species and their chromosomal distribution in four *Allium* species. Journal of Plant Biology 55: 15–25. 10.1007/s12374-011-9185-4

[B162] Stack SM Roelofs D (1996) Localized chiasmata and meiotic nodules in the tetraploid onion *Alliumporrum*. Genome 39: 770–783. 10.1139/g96-097 18469935

[B163] Sulistyaningsih E Yamastai K Tashiro Y (2002) Genetic characteristics of the Indonesian white shallot. Journal of the Japanese Society for Horticultural Science 71: 504–508. 10.2503/jjshs.71.504

[B164] Sýkorová E Leitch AR Fajkus J (2006) Asparagales telomerases which synthesize the human type of telomeres. Plant Molecular Biology 60(5): 633–646. 10.1007/s11103-005-5091-9 16649103

[B165] Sýkorová E Lim KY Kunická Z Chase MW Bennett MD Fajkus J Leitch AR (2003) Telomere variability in the monocotyledonous plant order Asparagales. Proceedings of the Royal Society of London, Series B, Biological Sciences 270(1527): 1893–1904. 10.1098/rspb.2003.2446 PMC1691456 14561302

[B166] Talukder K Sen S (1999) *In situ* cytophotometric estimation of nuclear DNA in different cultivars of *Allium* species. Cytobios 99(390): 57–65.

[B167] Talukder K Sen S (2000) Chromosome characteristics in some *Allium* sp. and assessment of their interrelationship. The Nucleus 43(1, 2): 46–57.

[B168] Tanaka R Taniguchi K (1975) A banding method for plant chromosomes. Japanese Journal of Genetics 50: 163–167. 10.1266/jjg.50.163

[B169] Tang H Lihua M Shiqimg A Jianquaan L (2005) Origin of the Qinghai-Tibetan Plateau endemic *Milula* (Liliaceae): further insights from karyological comparisons with *Allium*. Caryologia 58(4): 320–331. 10.1080/00087114.2005.10589470

[B170] Tardif B Morisset P (1992) Relation between numbers of B-chromosomes and C-bands in *Alliumschoenoprasum* L. Cytologia 57: 349–352. 10.1508/cytologia.57.349

[B171] Ulrich I Fritz B Ulrich W (1988) Application of DNA fluorochromes for flow cytometric DNA analysis of plant protoplasts. Plant Science 55: 151–158. 10.1016/0168-9452(88)90171-9

[B172] Vakhtina LI Zakirova RO Vakhtin YB (1977) Interspecific differences in DNA contents and taxonomically valid characters in *Allium* L. (Liliaceae). Botanicheskii Zhurnal (Moscow & Leningrad) 62: 667–684.

[B173] Van-Lume B Esposito T Diniz-Filho JAF Gagnon E Lewis GP Souza G (2017) Heterochromatic and cytomolecular diversification in the *Caesalpinia* group (Leguminosae): relationships between phylogenetic and cytogeographical data. Perspectives in Plant Ecology, Evolution and Systematics 29: 51–63. 10.1016/j.ppees.2017.11.004

[B174] Van‘t Hof J (1965) Relationships between mitotic cycle duration, S period duration and the average rate of DNA synthesis in the root meristem cells of several plants. Experimental Cell Research 39: 48–58. 10.1016/0014-4827(65)90006-6 5831250

[B175] Ved Brat S (1965) Genetic systems in *Allium*. I. Chromosome variation. Chromosoma 16: 486–499. 10.1007/BF00343176

[B176] Ved Brat S (1967) Genetic systems in *Allium* IV. Balance in hybrids. Heredity 22: 387–396. 10.1038/hdy.1967.48

[B177] Ved Brat S Dhingra B (1973) Genetic systems in *Allium* V. Breakdown of classical system in *Alliumcepa*. The Nucleus 16: 11–19.

[B178] Vitales D D’Ambrosio U Galvez F Kovařík A Garcia S (2017) Third release of the plant rDNA database with updated content and information on telomere composition and sequenced plant genomes. Plant Systematics and Evolution 303(8): 1115–1121. 10.1007/s00606-017-1440-9

[B179] von Bothmer R (1975) The *Alliumampeloprasum* complex on Crete. Mitteilungen (aus) der Botanischen Staatssammlung München 12: 267–288.

[B180] Vujošević M Jovanović V Blagojević J (2013) Polyploidy and B chromosomes in *Alliumflavum* from Serbia. Archives of Biological Sciences 65(1): 23–32. 10.2298/ABS1301023V

[B181] Wajahatullah MK Vahidy AA (1990) Karyotyping and localization of nucleolar organizer regions in garlic, *Alliumsativum* L. Cytologia 55: 501–504. 10.1508/cytologia.55.501

[B182] Walters ZW (1992) Rapid nuclear DNA content estimation for *Allium* spp. using flowcytometry. Allium Improvement Newsletter 2: 4–6.

[B183] Wang C Zheng G (1987) The relationship between the intercellular chromatin migration of pollen mother cells and the changes of chromosome numbers during the genesis of male gametes in *Alliumcepa*. Acta Botanica Sinica 29: 247–252.

[B184] Weiss H Scherthan H (2002) *Aloe* spp. – plants with vertebrate-like telomeric sequences. Chromosome Research 10(2): 155–164. 10.1023/A:1014905319557 11993936

[B185] Wufeng D Jinqiao X Xin X (1993) Studies on karyotypes of four Chinese scallions (*Alliumchinensis* G. Don). Journal of Wuhan Botanical Research 11: 199–203.

[B186] Xie-Kui C Ao C Zhang Q Chen L Liu J (2008) Diploid and tetraploid distribution of *Alliumprzewalskianum* Regel. (Liliaceae) in the Qinghai-Tibetan Plateau and adjacent regions. Caryologia 61(2): 192–200. 10.1080/00087114.2008.10589629

[B187] Xu J Yang L He X Xue P (1998) A study on karyotype differentiation of *Alliumfasciculatum* (Liliaceae). Acta Phytotaxonomica Sinica 36(4): 346–352.

[B188] Xue CY Xu JM Liu JQ (2000) Karyotypes of nine populations of *Alliumprzewalskianum* from Qinghai. Acta Botanica Yunnanica 22: 148–154.

[B189] Yang L Xu J Zhang X Wan H (1998) Karyotypical studies of six species on the genus *Allium*. Acta Phytotaxonomica Sinica 36(1): 36–46.

[B190] Yuzbasioglu D (2004) Karyotyping, C- and NOR banding of *Alliumsativum* L. (Liliaceae) cultivated in Turkey. Pakistan Journal of Botany 36(2): 343–349.

[B191] Zakirova RO Nafanailova II (1988) Chromosome numbers in some species of the Kazakhstan flora. Botanicheskii Zhurnal (Moscow & Leningrad) 73: 1493–1494.

[B192] Zhukova PG (1967) Karyology of some plants cultivated in the Arctic-Alpine Botanical Garden. In: Avrorin NA (Ed.) Plantarum in Zonam Polarem Transportatio II.Leningrad, 139–149.

